# One‐year outcomes after prostate artery embolization versus laser enucleation: A network meta‐analysis

**DOI:** 10.1002/bco2.302

**Published:** 2023-10-27

**Authors:** Ansh Bhatia, Joao Gabriel Porto, Aneesha Maini, Deepak Langade, Thomas R. W. Herrmann, Hemendra Navinchandra Shah, Shivank Bhatia

**Affiliations:** ^1^ Department of Interventional Radiology, Miller School of Medicine University of Miami Miami Florida USA; ^2^ Seth GS Medical College and KEM Hospital Mumbai India; ^3^ Department of Urology University of Miami Miami Florida USA; ^4^ School of Medicine Georgetown University Washington District of Columbia USA; ^5^ School of Medicine DY Patil University Navi Mumbai India; ^6^ Department of Urology Switzerland Urology Spital Thurgau AG (STGAG) Frauenfeld Switzerland; ^7^ Department of Urology, Desai Sethi Urology Institute, Miller School of Medicine University of Miami Miami Florida USA

**Keywords:** benign prostatic hyperplasia, complications, endoscopic enucleation of prostate, enlarged prostate, holmium laser enucleation of prostate, minimally invasive surgical therapy, prostate artery embolization

## Abstract

**Background:**

Although holmium laser enucleation (HoLEP) is considered a size‐independent procedure for treatment of an enlarged prostate, prostate artery embolization (PAE) is emerging as an alternative modality to treat moderate and large benign prostatic hyperplasia. This study aims to compare the early post‐operative and short‐term efficacy of PAE and HoLEP.

**Methods:**

PubMed, Cochrane Library and EMBASE databases were searched. Network meta‐analysis was performed following PRISMA‐N‐guidelines. Post‐operative parameters analysed include international prostate symptom score (IPSS), quality of life (QOL), post‐void residual urine (PVR), maximal uroflow rate (Qmax) and serious adverse events (SAE). Random effects model calculated weighted mean differences (WMD). If 95%CI crossed the line of no effect (WMD = 0), evidence indicated no statistically significant difference between treatments compared.

**Results:**

Qualitative and quantitative syntheses included 20 and 18 studies with 1991 and 1606 patients, respectively. At 3 months, there was no statistically significant difference between PAE and HoLEP in IPSS score improvement [WMD: −2.21: 95%CI: (−10.20, 5.78), favouring PAE], QoL score improvement [WMD: −0.22:95%CI: (−1.75, 1.32), favouring PAE] and PVR improvement [WMD: 26.97: 95%CI: (−59.53, 113.48), favouring HoLEP]. However, PAE was found inferior to HoLEP for Qmax improvement [WMD: 8.47, 95%CI: (2.89, 14.05), favouring HoLEP]. At 1‐year follow‐up, there was no statistically significant was found between PAE and HoLEP for IPSS score improvement [WMD:6.03, 95%CI: (−1.30, 13.35)], QoL score improvement [WMD: 0.03, 95%CI: (−1.19, 1.25)], PVR improvement [WMD:4.11, 95%CI: (−32.31, 40.53)] and Qmax improvement [WMD:2.60, 95%CI: (−2.20, 7.41)] with all differences favouring HoLEP. PAE was superior to HoLEP for SAE [PAE vs. HoLEP‐OR: 0.68, 95%CI: (0.25, 1.37)].

**Conclusion:**

HoLEP was superior to PAE at 3 months for Qmax improvement. There was no significant difference in IPSS, QoL, PVR and Qmax improvement at 1 year between PAE and HoLEP. PAE was also associated with lesser SAE compared to HoLEP. Studies on the long‐term outcome of PAE are needed to establish the durability of early outcomes after PAE.

## 1 INTRODUCTION

Lower urinary tract symptoms (LUTS) secondary to benign prostatic hyperplasia (BPH) can significantly impact patients' quality of life.^1^ Transurethral resection of prostate (TURP) is currently considered the gold standard of BPH treatment.^2^ Recently, minimally invasive surgical therapies (MIST) for treating BPH have gained popularity in reducing morbidity inherent to TURP. These include Urolift, REZUM, prostatic stents and prostate artery embolization (PAE).^3^ PAE has been shown to provide significant symptom improvement in both large‐ and medium‐sized prostates.^4^, ^5^ Other situations where PAE may be suitable are when the patient is unfit/unwilling to undergo surgery or concerns about adverse events due to its minimally invasive nature.

Similarly, endoscopic enucleation has been offered as a less invasive alternative to TURP. Holmium laser enucleation (HoLEP) is considered a size‐independent procedure to surgically treat an enlarged prostate by the American Urological Association (AUA) and the European Association of Urology (EAU) guidelines,^6^, ^7^, ^8^, especially in high‐risk surgical candidates and those on anticoagulants.

Although PAE and HoLEP have been individually compared to TURP in various individual and pooled analyses,^9^, ^10^ and network meta‐analyses (NMAs) have summarised various surgical options,^9^ there is no NMA comparing PAE and HoLEP. Both these procedures could be offered to patients with BPH, those at elevated risk for surgery and those on anticoagulants. In addition, both have different post‐operative recovery with complication rates. Therefore, it is necessary to compare the outcomes of these two procedures. This study aims to answer the question: Is there a superior treatment with respect to efficacy and safety between PAE and HoLEP for the treatment of men with moderate to severe LUTS due to BPH at 3 months and 1 year?

## 2 MATERIALS AND METHODS

This NMA was registered to the PROSPERO database (registration number PROSPERO‐2022‐CRD42022339240) and was conducted following the PRISMA‐N guidelines (Figure S1A). A search of Medline, EMBASE and the Cochrane Library database was conducted from inception to July 2022 with keywords (and combinations thereof) such as ‘Prostate artery embolization’, ‘Holmium Laser enucleation’, ‘Transurethral resection’ and ‘Benign Prostatic Hyperplasia’ and Boolean operators were used.

Given that TURP is the gold standard of BPH treatment and the recommended use of TURP for both medium and large prostates,^3^ TURP was considered as the standard comparator node between PAE and HoLEP. Prospective, parallel group randomised controlled trials that compared the effect of PAE and TURP/Sham or HoLEP and TURP/Sham and had at least one of the following outcome variables: IPSS, IPSS‐QoL score, PVR, and Qmax at least one of the following time points: 3, 6, 12 and 24 months were included in the quantitative synthesis. Non‐randomized controlled trials, studies without even one of the outcome variables, studies with different indications for treatments under investigation, cost analysis and case studies were excluded from the NMA. Studies with different indications such as PAE for prostate cancer–associated hematuria or re‐intervention after surgery for BPH. Similarly, different populations such as men with concomitant multiple LUTS treatment, LUTS due to prostate cancer and bladder neck/urethral stricture were excluded. The PICO criteria were used to identify eligible studies, as shown in Figure S2. Bipolar TURP and Monopolar TURP were combined in the same node due to similar efficacies and this study's indirect comparison (PAE vs. HoLEP) nature. The complete search strategy for each database can be found in the supplemental dataset (Figure S1B). The study selection process is depicted in Figure S3. After the search results were exported to Excel, duplicates were removed via software, and studies were screened based on the title and abstract. Screened studies underwent full‐text review by two authors (AB and AM), and disagreement was resolved with consensus and input of a third member (SB). The two authors (AB and AM) extracted the data from the selected studies, and the extraction was cross‐verified. Data points for 3 and 12 months were utilized for quantitative synthesis of early post‐operative and short‐term follow‐up, respectively. If the data for these time points were unavailable, the closest follow‐up data were used. The geometry of the network is represented in Figure S4. Risk of bias and assessment was done by the two authors (AB and AM) using the NIH quality assessment tool to evaluate the studies' quality.^11^ The studies were rated as ‘Good’, ‘Fair’ or ‘Bad’ according to the NIH criteria (Tables 2A, 2B and 2C).

### 2.1 Statistical analysis and summary measures

The analysis used MetaXL Version 5.3 (Epigear International Pty Ltd, Australia). Generalized pairwise modelling (GPM) was implemented for this NMA since it requires fewer assumptions than the Frequentist or Bayesian methods. Dichotomous outcomes (adverse events) were analysed by risk ratio (RR) with 95% confidence intervals (CIs), and continuous outcomes were analysed according to weighted mean difference (WMD) with 95%CIs to estimate the effect size. A visual inspection of forest plots assessed heterogeneity and subsequently tested using the I^2^ statistic. The random effects model was used wherever high heterogeneity was present. In addition, Cochrane's Q and consistency H were calculated. A traditional pairwise meta‐analysis was performed for the new interventions, PAE and HoLEP, compared independently with TURP. Statistical significance for the heterogeneity of the pooled estimate was set at *P* < 0.05. Treatments were considered statistically superior if the 95%CI did not cross the line of no effect. NMA was performed separately for each outcome to determine the treatment effect estimate. LFK index (3) was calculated to evaluate the risk of publication bias.

## 3 RESULTS

One hundred eight studies were assessed for eligibility. A total of 18 studies representing 1606 BPH patients were included in the quantitative synthesis,^12^, ^13^, ^14^, ^15^, ^16^, ^17^, ^18^, ^19^, ^20^, ^21^, ^22^, ^23^, ^24^, ^25^, ^26^, ^27^, ^28^, ^29^, ^30^ whereas 20 studies representing 1991 patients were included in the qualitative synthesis. The baseline characteristics of the studies are included in Tables 1A, 1B, 1C and 1D. The results of the quality assessment (Tables 2A and 2B) showed that two studies were rated ‘Good’, and the remaining studies were rated ‘Fair’. Table 2C shows the quality of evidence for each outcome. Table S1 contains technical details about the technique and equipment used for each procedure.

**TABLE 1A:** Demographic and study type data for PAE studies.

Study name (Country)	Type of study	Sample size	Imaging modality	PAE			TURP		
				BMI	Age	Prostate volume (mL)	BMI	Age	Prostate volume (mL)
Abt, 2021 (Switzerland) ^24^	RCT	102 51 (PAE) 52 (TURP)	MRI & TRUS	26.5 ± 44.2	65.7 ± 9.3	56.5 ± 31.1 (MRI) & 52.1 ± 18.6 (TRUS)	27.0 ± 3.9	66.1 ± 9.8	56.5 ± 31.1 (MRI) & 52.1 ± 18.6 (TRUS)
Abt, 2018 (Switzerland) ^30^	RCT	99 48 (PAE) 51 (TURP)	MRI & TRUS	26.5 ± 4.2	65.7 ± 9.3	51.2 ± 16.5	27.0 ± 3.9	66.1 ± 9.8	52.1 ± 18.6
Gao, 2014 (China) ^23^	RCT	114. 57 (PAE) 57 (TURP)	MRI & TRUS	NR	67.7 ± 8.7	64.7 ± 19.7	NR	66.4 ± 7.8	63.56 ± 18.6
Zhu, 2018 (China) ^31^	RCT	40 20 (PAE) 20 (TURP)	MRI & TRUS	NR	61.1 ± 4.4	81.21 ± 6.34	NR	62.4 ± 4.9	82.09 ± 6.47
Insausti, 2020 (Spain) ^32^	RCT	61 31 (PAE) 30 (TURP)	TRUS	72.4 ± 6.2	72.4 ± 6.2	60 ± 21.6	NR	71.8 ± 5.5	62.8 ± 23.8
Carnavale 2016 (Brazil) ^40^	RCT	30 15 (PAE) 15 (TURP)	MRI & TRUS	NR	63.5 ± (8.7)	63 ± (17.8)	NR	NR	56.6 ± 21.5
Radwan 2020 (Egypt) ^25^	RCT	60 20 (PAE) 40 TURP)	TRUS	NR	60 ± 22.6	26.7 ± (3.3)	NR	63	63.5 ± 22.6
Yoshinaga 2014 (Brazil) ^41^	RCT	30 15 (PAE) 15(TURP)	MRI	NR	63.1 ± 8.6	59.3 ± 16.7	NR	66.3 ± 5.8	57.6 ± 27.1
Pisco 2020 (Portugal) ^15^	RCT	80 40 (PAE) 40 (Sham)	MRI & TRUS	NR	64 (median) (59–67.5) (Q1–Q3)	63.5 (55.5–100.0)–TRUS 68.5 (58.0–103.5)–MRI	26.9 (25.5–27.9)	64 (median) (60–68.5) (Q1–Q3)	66 (median) (55.5–94.5) (Q1–Q3)
Ray 2018 (United Kingdom) ^18^, ^a^	Observational	305 216 (PAE) 89 (TURP)	MRI	NR	66 ± 7.4	101.2 ± 57.1	NR	70 ± 7.5	68.7 ± 9.2

*Note*: Notably, average prostate sizes in all studies are similar and represent medium‐sized BPH. All data are presented as mean ± SD unless otherwise specified.

Abbreviations: MRI, magnetic resonance imaging; NR, not reported; TRUS, transrectal ultrasound.

^a^ These observational studies are not part of the NMA but are included in the SR.

**TABLE 1B:** Demographic and study type data for HoLEP studies.

Study name (Country)	Type of study	Sample size	Imaging modality	HoLEP		TURP	
				Age	Prostate volume (mL)	Age	Prostate volume (mL)
Sun, 2014 (China) ^16^	RCT	164 82 (HoLEP), 82 (TURP)	TRUS	72.16 ± 7.53	55.11 ± 29.03	72.16 ± 7.53	56.22 ± 30.48
Fayad, 2015 (Egypt) ^33^	RCT	120 60 (HoLEP), 60 (TURP)	TRUS	60.85 ± 4.02	68.15 ± 11.16718	60.35 ± 3.926	67.2 ± 9.71945
Gilling, 2012 (New Zealand) ^34^	RCT	31 14 (HoLEP), 17 (TURP)	TRUS	71.7 ± 1.1	77.68 ± 32.12	70.3 ± 1	70 ± 27.78
Kuntz, 2004 (Germany) ^17^	RCT	200 100 (HoLEP), 100 (TURP)	TRUS	68.0 ± 7.3	53.5 ± 20.0	68.7 ± 8.2	49.9 ± 21.1
Gupta, 2005 (India) ^26^	RCT	150 50 (HoLEP), 100 (TURP)	TAUS	65.88 ± 10.1	57.9 ± 17.6	65.67 ± 7.5	59.8 ± 16.5
Montorsi, Rigatti, 2004 (Italy) ^35^	RCT	100 52 (HoLEP), 48 (TURP)	TRUS	65.14	70.3 ± 36.7	64.5	56.2 ± 19.4
Tan, 2003 (New Zealand)/Liam, 2006 ^36^	RCT	61 31 (HoLEP), 30 (TURP)	TRUS	71.7 ± 1.1	77.8 ± 5.6	70.3 ± 1	70 ± 5
Basic, 2013 (Serbia)^37^	RCT	40 20 (HoLEP), 20 (TURP)	TRUS	63.3 ± 7.4	48.8 ± 4.9	65.1 ± 6.9	42.6 ± 4.4
Fayad, 2011 (Egypt) ^38^	RCT	60 30 (HoLEP), 30 (TURP)	TRUS	60.0667 ± 4.51	76.6 ± 17.22218	61.2 ± 4.21	80.6 ± 17.78725
Jhanwar, 2016 (India) ^39^	RCT	144 72 (HoLEP), 72 (TURP)	TRUS	67.70 ± 7.44	75.6 ± 12.84	66.78 ± 7.81	74.5 ± 12.56

*Note*: Notably, average prostate sizes in all studies are similar and represent medium‐sized BPH. All data are presented as mean ± SD unless otherwise specified. No HoLEP study reported the BMI. So the BMI column was not represented here.

Abbreviation: NR, not reported.

**TABLE 1C:** DUC, hospitalization times and most common SAEs in PAE studies.

Study name (Country)	PAE			TURP		
	Duration under catheterization	Length of stay	Most common SAE^a^	Duration under catheterization	Length of stay	Most common SAE^a^
Abt, 2021 (Switzerland) ^24^	NR	NR	Acute urinary retention, Severe hematuria	NR	NR	Stricture, severe hematuria, urinary retention
Abt, 2018 (Switzerland) ^30^	1.3 ± 1.4 days	2.2 ± 0.6 days	Urinary retention, severe hematuria	3.3 ± 1.4 days	4.2 ± 1.7 days	Urinary incontinence, urinary retention, stricture
Gao, 2014 (China) ^23^	NR	2.9 ± 1.6	Clinical failure, technical failure	NR	4.8 ± 1.8	Stricture (2.1%), Bladder neck stenosis (2.1%), TUR syndrome
Zhu, 2018 (China) ^31^	NR	NR	None	NR	NR	Acute urinary retention
Insausti, 2020 (Spain) ^32^	1–2 days	1 (0) (median; IQR)	None	4–5 days	1 (1) days (median; IQR)	Urethral stricture
Carnavale 2016 (Brazil) ^40^	NR	6 h	None	NR	2.1 (2–3 days)	Rupture of prostatic capsule
Radwan 2020 (Egypt) ^25^	5 days	NR	NR	3 days	NR	NR
Yoshinaga 2014 (Brazil) ^41^	NR	NR	NR	NR	NR	NR

^a^ Only up to the three most common SAEs are displayed here. All values are mean ± SD unless otherwise specified.

**TABLE 1D:** DUC, hospitalization times and most common SAEs in HoLEP studies.

Study name (Country)	HoLEP			TURP		
	Duration under catheterization (mean ± SD)	Length of hospital stay (mean ± SD)	Most common SAE^a^	Duration under catheterization (mean ± SD)	Length of stay (mean ± SD)	Most common SAE^a^
Sun, 2014 (China) ^16^	113.63 ± 50.61 h	11.37 ± 3.39 days	NR	127.43 ± 75.93 h	11.82 ± 3.41 days	NR
Fayad, 2015 (Egypt) ^33^	24–72 h	NR	NR	48–72 h	NR	NR
Kuntz, 2004 (Germany) ^17^	27.6 ± 10.4 h	53.3 ± 15.9 h	Bladder neck contracture, urethral stricture.	43.4 ± 21.1 h	85.8 ± 39.1 h	Bladder neck contracture, urethral stricture.
Gupta, 2005 (India) ^26^	28.6 ± 20.5 h	NR	Re‐catheterization, urethral stricture	45.7 ± 12.7 h	NR	Re‐catheterization, urethral stricture, TUR syndrome
Montorsi, Rigatti, 2004 (Italy) ^35^	31 ± 13 h	59 ± 19.9 h	Urethral stricture, AUR, severe bleeding	57.78 ± 17.5 h	85.8 ± 18.9 h	Urethral stricture, TUR syndrome, AUR
Tan, 2003 (New Zealand)/Liam, 2006 ^36^	17.7 ± 0.7 h	27.6 ± 2.7 h	Re‐catheterization, urethral stricture	Mean: 44.9 ± 10.1 Hours	Mean: 49.9 ± 5.6 Hours	Re‐catheterization, urethral stricture
Basic, 2013 (Serbia) ^37^	2.6 ± 3.8 days	3.1 ± 3.8 days	AUR, prolonged urinary incontinence	4.2 ± 3.6 days	4.4 ± 3.9 days	AUR, bladder neck stricture
Fayad, 2011 (Egypt) ^38^	NR	Mean: 49.06 ± 5.84 h	NR	NR	Mean: 80 h	NR
Jhanwar, 2016 (India) ^39^	30.94 ± 5.49 h	41.81 ± 9.17 h	Urethral stricture	48.06 ± 13.36 h	54.58 ± 12.36 h	None

^a^ Only up to the three most common SAEs are displayed here. All values are mean ± SD unless otherwise specified.

**TABLE 2A:** NIH quality assessment tool used to quantify risk of bias in individual PAE studies.

Study	NIH Q 1	Q2	Q 3	Q 4	Q 5	Q 6	Q 7	Q 8	Q 9	Q 10	Q 11	Q 12	Q 13	Q 14	NIH scale rating
Abt D et al. (2021/2018)	Y	Y	N	N	N	Y	N	N	Y	Y	Y	Y	N	N	Fair
Insausti I et al. (2020)	Y	Y	NR	N	N	Y	Y	Y	Y	CD	Y	N	Y	N	Fair
Radwan A et al. (2020)	Y	CD	N	N	N	Y	Y	Y	Y	NR	Y	NR	Y	NR	Fair
Carnevale FC et al. (2016)	Y	Y	N	N	N	Y	Y	Y	Y	Y	Y	N	Y	Y	Fair
Gao YA et al. (2014)	Y	Y	Y	N	N	Y	Y	Y	Y	Y	Y	Y	Y	N	Good
Zhu, C. et al. (2018)	Y	Y	N	N	N	Y	N	Y	Y	NR	Y	Y	NR	N	Fair
Yoshinaga EM et al. (2014)	Y	NR	NR	NR	NR	Y	Y	Y	Y	NR	Y	Y	N	Y	Fair
Ray et al. (2018)^a^	Y	Y	Y	N	Y	CD	Y	N	N	Y	Y	N	CD	N	Fair
Soluyanov et al. (2018)^b^	Y	Y	Y	N	Y	Y	N	Y	Y	Y	N	NA	‐	‐	Fair

^a^ NIH quality assessment tool for observational studies.

^b^ NIH quality assessment tool for before–after (pre–post) studies with no control group.

**TABLE 2B:** Results of the NIH quality assessment tool for individual HoLEP studies.

Study	NIH Q 1	Q 2	Q 3	Q 4	Q 5	Q 6	Q 7	Q 8	Q 9	Q 10	Q 11	Q 12	Q 13	Q 14	Rating
Sun N et al. (2014)	Y	Y	Y	Y	Y	Y	Y	Y	Y	NR	Y	NR	Y	CD	Good
Fayad et al. (2015)	Y	N	N	N	N	Y	Y	Y	Y	NR	Y	Y	Y	N	Fair
Kuntz et al. (2004)	Y	N	N	NR	N	Y	Y	Y	Y	NR	Y	Y	NR	Y	Fair
Gupta N et al. (2005)	Y	NR	NR	NR	NR	Y	NR	NR	Y	NR	Y	NR	NR	Y	Fair
Wilson et al. (2006)	Y	NR	NR	NR	NR	Y	Y	N	Y	NR	Y	N	NR	N	Fair
Montorsi, Rigatti et al. (2004)	Y	NR	NR	NR	NR	Y	Y	Y	Y	NR	Y	NR	NR	Y	Fair
Basić D et al. (2013)	Y	NR	NR	NR	NR	Y	Y	Y	Y	NR	Y	NR	Y	Y	Fair
Tan AH et al. (2003)/Gilling et al. (2012)	Y	Y	Y	N	N	Y	Y	Y	Y	NR	Y	N	NR	Y	Fair
Fayad AS et al. (2011)	Y	NR	NR	N	N	Y	Y	Y	Y	NR	Y	N	Y	Y	Fair
Jhanwar et al. (2006)	Y	Y	Y	N	N	Y	Y	Y	Y	NR	Y	CD	Y	N	Fair

**TABLE 2C:** Quality of evidence for each outcome.

Study outcome	Quality of evidence	
	3 months	1 year
IPSS	Good: 1 study	Good: 2 studies
	Fair: 10 studies	Fair: 11 studies
QoL	Good: 1 study	Good: 2 studies
	Fair: 7 studies	Fair: 7 studies
Qmax	Good: 2 studies	Good: 2 studies
	Fair: 10 studies	Fair: 11 studies
PVR	Good: 2 studies	Good: 2 studies
	Fair: 6 studies	Fair: 7 studies
SAE	Good: 1 study	
	Fair: 12 studies	

*Note*: This table shows the quality of the studies included for each metric in the network. Please refer to Table 2A and 2B for the quality analysis of individual studies.

### 3.1 Quantitative analyses

#### 3.1.1 IPSS (Figure 1A,B)

**FIGURE 1:**
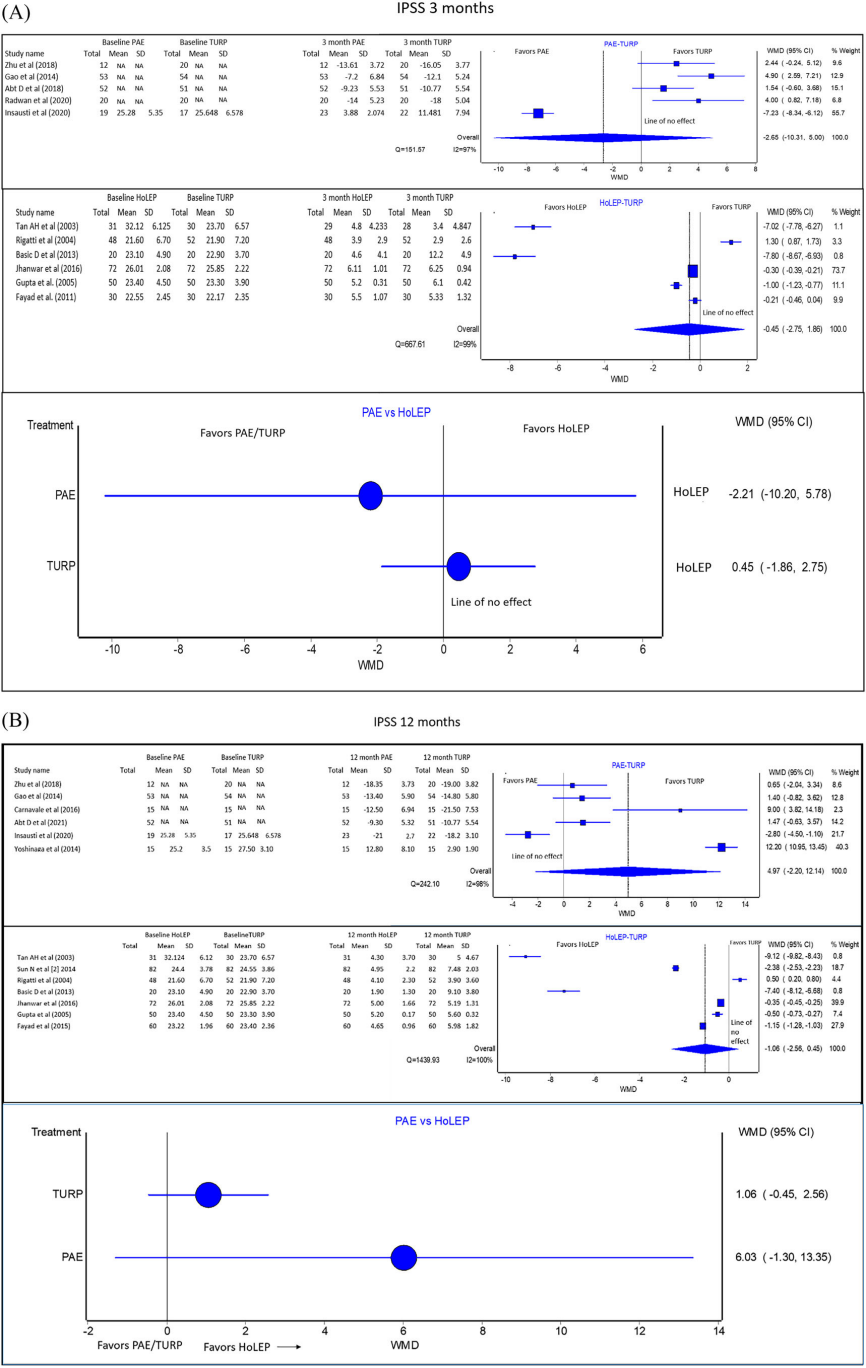
(A) (From top to bottom): Pooled IPSS analysis results of PAE versus TURP, HoLEP versus TURP and PAE versus HoLEP (indirect comparison) at 3 months. If the 95% CI crosses this line, the evidence does not support one treatment over the other. These studies have directly reported change from baseline. Figure 1 shows the results of the network meta‐analysis, divided by time points and treatment outcomes. (B) (From top to bottom): Pooled IPSS analysis results of PAE versus TURP, HoLEP versus TURP and PAE versus HoLEP (indirect comparison) at 12 months. If the 95% CI crosses this line, the evidence does not support one treatment over the other. These studies have directly reported change from baseline. NA, not available.

At 3 months, there was no statistically significant difference between PAE and HoLEP compared to TURP for improvement in the IPSS score [PAE vs. TURP WMD: −2.65 with 95%CI: (−10.31 to 5.00) favouring PAE. 95%CI crosses the line of no effect–no statistically significant difference. HoLEP vs. TURP WMD: −0.45 with 95%CI: (−2.75 to 1.86) favouring HoLEP. 95%CI crosses the line of no effect–no statistically significant difference]. For the same time‐point, there was no statistically significant difference between PAE and HoLEP [WMD: −2.20 with 95%CI: (−10.20 to 5.78) in favour of PAE. 95% CI crosses the line of no effect–no statistically significant difference between treatments compared]. Quality of evidence: Fair.

At 1 year, there was no statistically significant difference between PAE and HoLEP compared to TURP for improvement in the IPSS score [PAE vs. TURP WMD: 4.97 with 95%CI: (−2.20 to 12.14) favouring TURP, HoLEP versus TURP WMD: −1.06 with 95%CI: (−2.56, 0.45) favouring HoLEP. 95%CI crosses the line of no effect–no statistically significant difference].

For the same duration, there is no statistically significant difference between PAE and HoLEP for improvement in IPSS score [Indirect comparison WMD: 6.03 95%CI: (−1.30 to 13.35) in favour of HoLEP. 95%CI crosses the line of no effect–no statistically significant difference in treatments compared]. Quality of evidence: Fair.

#### 3.1.2 QoL (Figure 2A,B)

**FIGURE 2:**
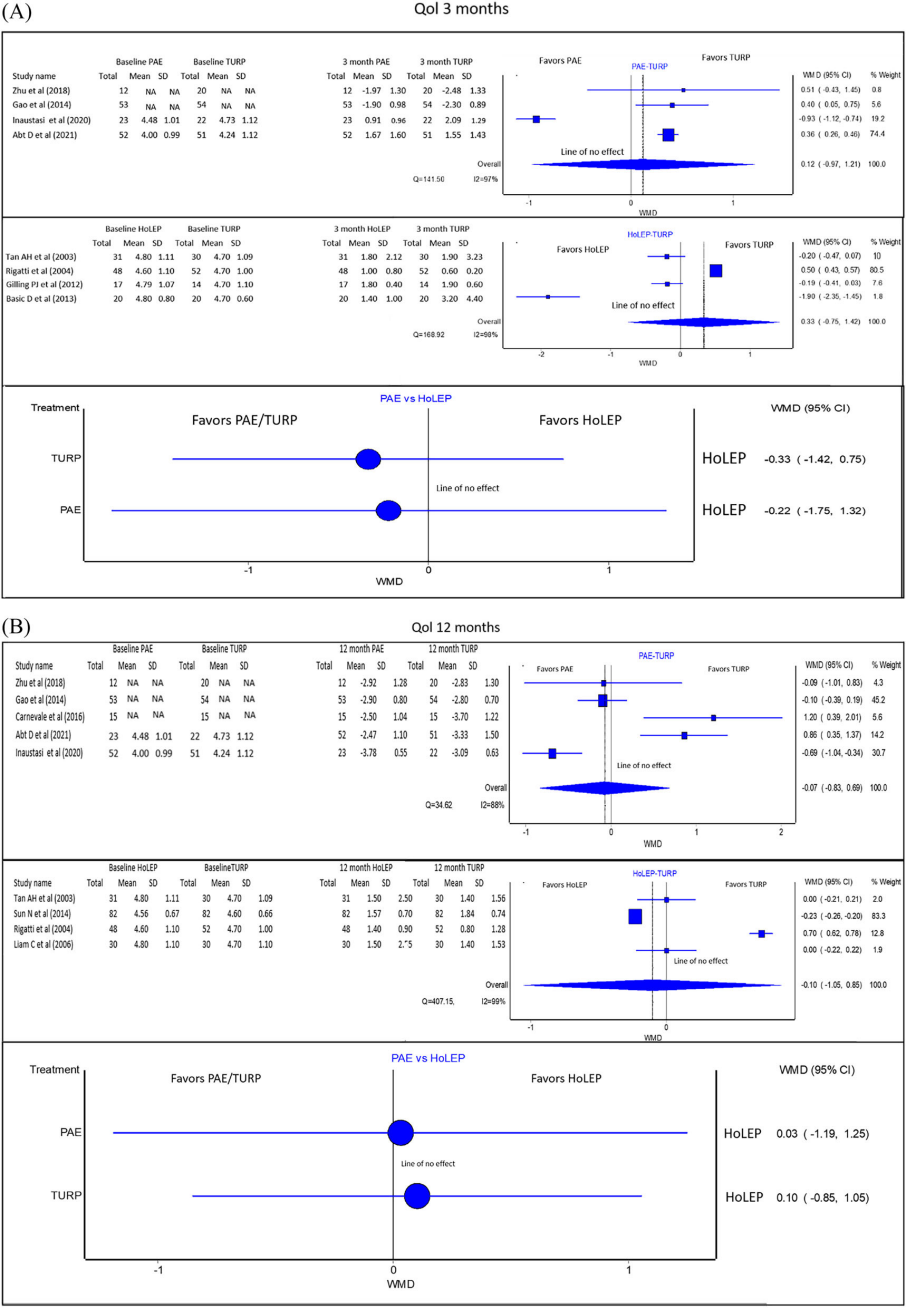
(A) (From top to bottom): Pooled QoL analysis results of PAE versus TURP, HoLEP versus TURP and PAE versus HoLEP (indirect comparison) at 3 months. If the 95%CI crosses the line of no effect, the evidence does not support one treatment over the other. These studies have directly reported change from baseline. (B) (From top to bottom): Pooled QoL analysis results of PAE versus TURP, HoLEP versus TURP and PAE versus HoLEP (indirect comparison) at 12 months. If the 95%CI crosses the line of no effect, the evidence does not support one treatment over the other. These studies have directly reported change from baseline. NA, not available.

At 3 months, there was no statistically significant difference between PAE and HoLEP compared to TURP for improvement in QoL score [PAE vs. TURP WMD: 0.12 with 95%CI: (−0.97 to 1.21) favouring TURP. 95%CI crosses the line of no effect–no statistically significant difference between treatments compared. HoLEP vs. TURP WMD: 0.33 with 95%CI: (−0.75 to 1.42) favouring TURP. 95%CI crosses the line of no effect–no statistically significant difference between treatments compared]. There is no statistically significant difference between PAE and HoLEP for improvement in QoL score for the same duration. [WMD: −0.22 with 95%CI: (−0.75 to 1.42) favouring PAE. 95%CI crosses the line of no effect–no statistically significant difference between treatments compared]. Quality of evidence: Fair.

At 12 months, there was no statistically significant difference; PAE and HoLEP are compared to TURP for improvement in QoL score [PAE vs. TURP WMD: −0.07 with 95%CI: (−0.83 to 0.69) in favour of PAE. HoLEP vs. TURP WMD: −0.10 with 95%CI: (−1.05 to 0.85) in favour of HoLEP. 95%CI crosses the line of no effect–no statistically significant difference between treatments compared in both comparisons]. For the same duration, there is no statistically significant difference between PAE and HoLEP for improvement in the QoL score. [Indirect comparison WMD: 0.03 with 95%CI: (−1.19 to 1.25) in favour of HoLEP. 95%CI crosses the line of no effect–no statistically significant difference between treatments compared]. Quality of evidence: Fair.

#### 3.1.3 PVR (Figure 3A,B)

**FIGURE 3:**
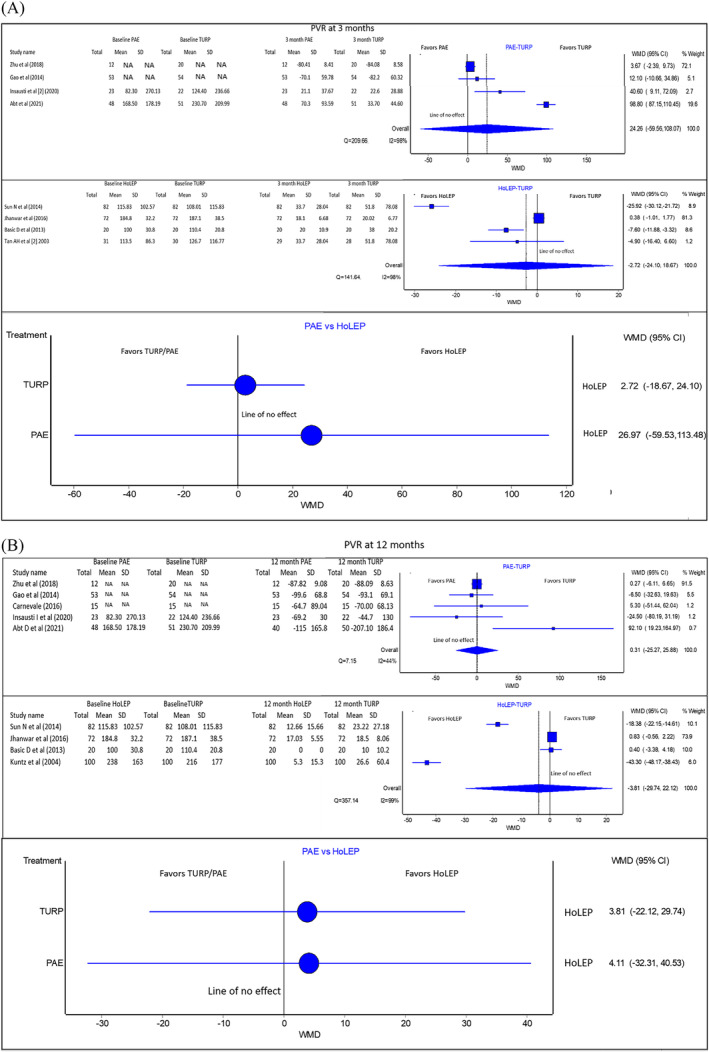
(A) (From top to bottom): Pooled PVR analysis results of PAE versus TURP, HoLEP versus TURP and PAE versus HoLEP (indirect comparison) at 3 months. If the 95% CI crosses the line of no effect, the evidence does not support one treatment over the other. These studies have directly reported change from baseline. (B) (From top to bottom): Pooled PVR analysis results of PAE versus TURP, HoLEP versus TURP and PAE versus HoLEP (indirect comparison) at 12 months. If the 95%CI crosses the line of no effect, the evidence does not support one treatment over the other. These studies have directly reported change from baseline. NA, not available.

At 3 months, there is no statistically significant difference between PAE and HoLEP compared to TURP for improvement in PVR [PAE vs. TURP WMD: 24.26 with 95%CI: (−59.56 to 108.07) favouring TURP. 95%CI crosses the line of no effect–no statistically significant difference between treatments compared. HoLEP vs. TURP WMD: −2.72 with 95%CI: (−24.10 to 18.67) favouring TURP. 95%CI crosses the line of no effect–no statistically significant difference between treatments compared]. For the same time‐point, there is no statistically significant difference between PAE and HoLEP for improvement in PVR volume [WMD 26.97 with 95%CI: (−59.53 to 113.48) in favour of HoLEP. 95%CI crosses the line of no effect–no statistically significant difference between treatments compared]. Quality of evidence: Fair.

At 12 months, there was no statistically significant difference between PAE and HoLEP compared to TURP for improvement in PVR. [PAE vs. TURP WMD: 0.31 with 95%CI: (−25.27 to 25.88) in favour of PAE. HoLEP vs. TURP WMD: −3.81 with 95%CI: (−29.74 to 22.12) in favour of HoLEP. In both cases, the 95%CI crosses the line of no effect no statistically significant difference between treatments compared]. For the same time‐point, no statistically significant difference between PAE and HoLEP [Indirect comparison WMD: 4.11 with 95%CI: (−32.31 to 40.53) in favour of HoLEP. 95%CI crosses the line of no effect–no statistically significant difference between treatments compared]. Quality of evidence: Fair.

#### 3.1.4 Qmax (Figure 4A,B)

**FIGURE 4:**
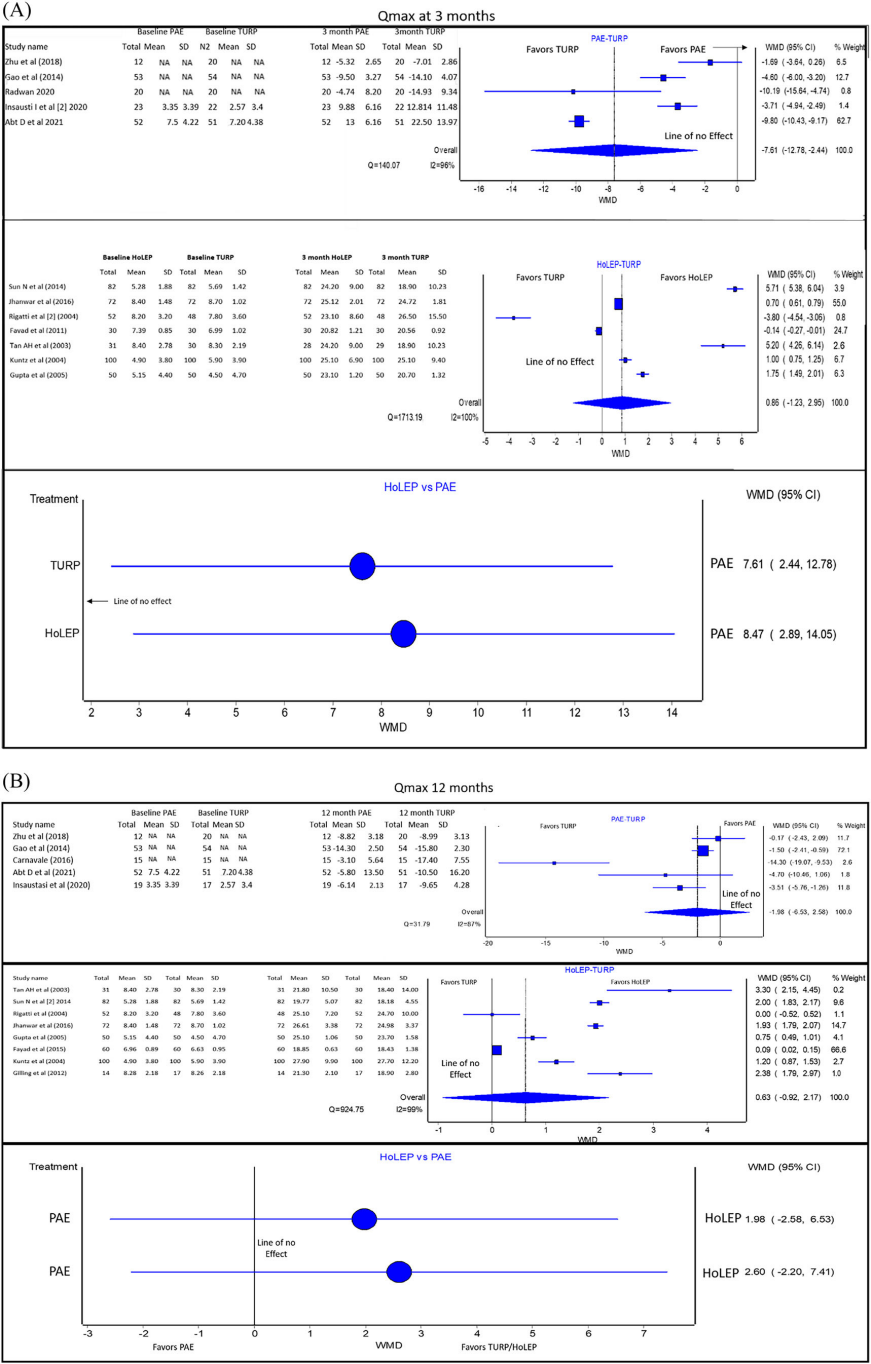
(A) (From top to bottom): Pooled Qmax analysis results of PAE versus TURP, HoLEP versus TURP and PAE versus HoLEP (indirect comparison) at 3 months. If the 95%CI crosses this line, the evidence does not support one treatment over the other. NA, not available. These studies have directly reported change from baseline. (B) (From top to bottom): Pooled analysis results of PAE versus TURP, HoLEP versus TURP and PAE versus HoLEP (indirect comparison) for Qmax at 12 months. If the 95% CI crosses the line of no effect, the evidence does not support one treatment over the other. These studies have directly reported change from baseline. NA, not available.

At 3 months, PAE is inferior to TURP for Qmax, whereas there was no statistically significant difference between HoLEP compared to TURP for improvement in Qmax [PAE vs. TURP WMD: −7.61 with 95%CI: (−12.78 to −2.44) favouring TURP. 95%CI does not cross the line of no effect–inferiority of one of the treatments compared. HoLEP vs. TURP WMD: 0.86 with 95%CI: (−1.23 to 2.95) favouring HoLEP. 95%CI crosses the line of no effect–no statistically significant difference]. For the same duration, there is a significant difference between PAE and HoLEP for improvement in Qmax [WMD: 8.47 with 95%CI: (2.88 to 14.05) in favour of HoLEP. 95%CI: does not cross the line of no effect–inferiority of treatments compared]. Quality of evidence: Good.

At 12 months, there was no statistically significant difference between PAE and HoLEP are compared to TURP for improvement in Qmax. [PAE vs. TURP WMD: −1.98 with 95%CI: (−6.53 to 2.58) in favour of TURP. HoLEP vs. TURP WMD: 0.63 with 95%CI: (−0.91 to 2.17) in favour of HoLEP. In both cases, the 95%CI crosses the line of no effect–no statistically significant difference between treatments compared]. For the same duration, there is no statistically significant difference between PAE and HoLEP for improvement in Qmax [Indirect comparison WMD: 2.60 with 95%CI: (−2.20 to 7.41) in favour of HoLEP. 95%CI crosses the line of no effect–no statistically significant difference of treatments compared]. Quality of evidence: Good.

#### 3.1.5 Serious adverse events (Figure 5)

**FIGURE 5:**
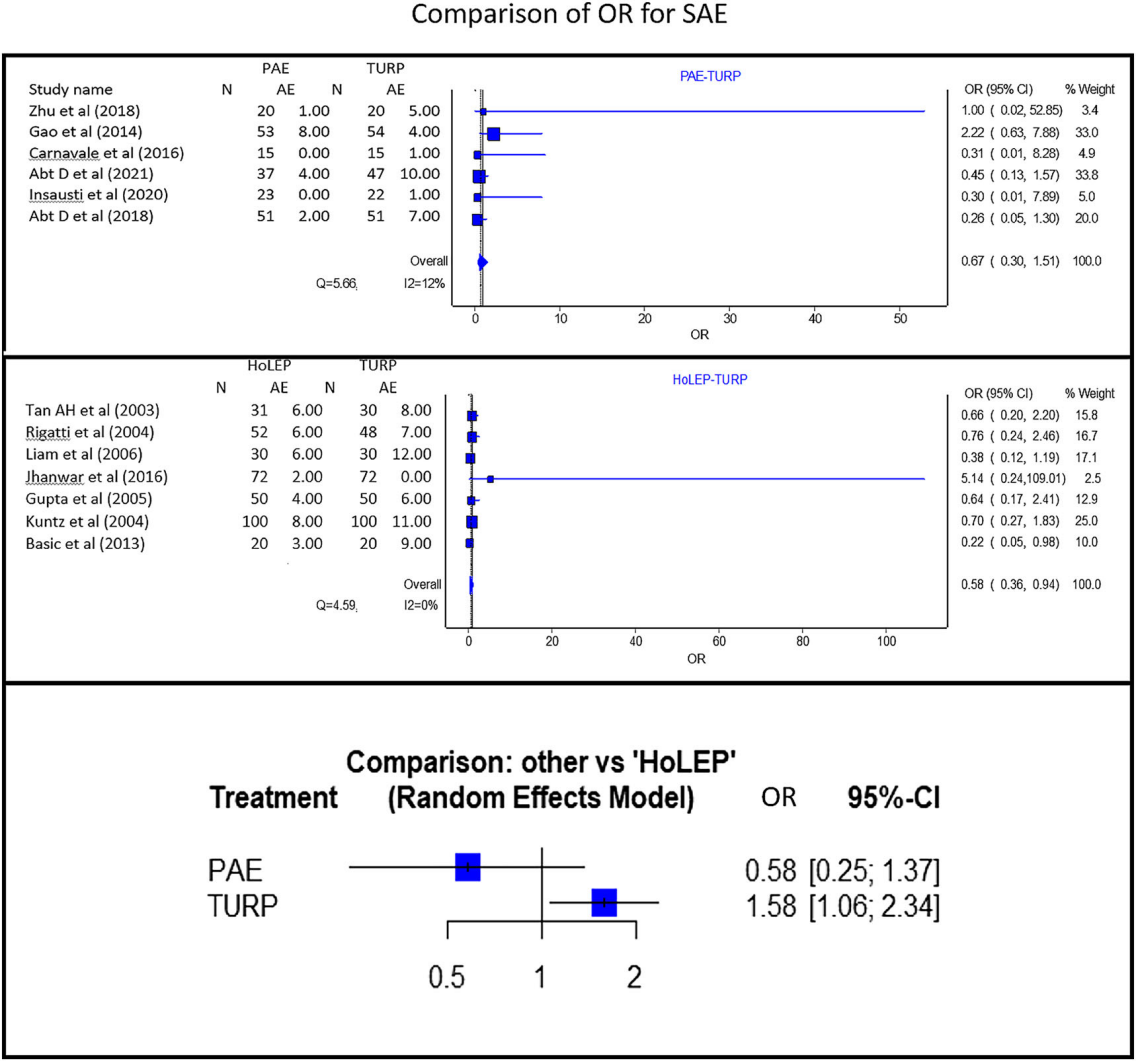
(From top to bottom): Pooled SAE analysis results of PAE versus TURP, HoLEP versus TURP and PAE versus HoLEP (indirect comparison) for SAE. If the 95% CI crosses the line of no effect, the evidence does not support one treatment over the other. These studies have directly reported change from baseline. NA, not available.

Serious adverse events (SAE) were defined as Clavien–Dindo level 3 and above. Although there was no statistically significant difference between PAE, HoLEP and TURP‐related SAE, PAE did show a lower SAE rate than TURP and HoLEP [PAE vs. HoLEP OR: 0.58 with 95%CI: (0.25 to 1.37), PAE showing fewer SAE]. PAE also showed fewer SAE than TURP [OR: 0.67 with 95%CI: (0.3 to 1.51). 95%CI crosses the line of no effect–no statistically significant difference of treatments compared]. HoLEP had significantly lower SAE rates compared to TURP. [TURP to HOLEP OR: 1.58, 95%CI: (1.06 to 2.34). 95%CI does not cross the line of no effect–statistically significant difference between treatments compared]. Quality of evidence: Fair.

### 3.2 Qualitative analysis results

The studies included in the quantitative synthesis (RCTs) were reviewed. Several trends were recorded in the review of studies. Most studies in the PAE group were from Western countries (USA, Western Europe) and China. The majority of HoLEP studies were also from Western countries, whereas some were from other regions such as India (two studies), Egypt (two studies) and Serbia (one study).

Although the inclusion criteria were varied (Table S1), all trials excluded patients with kidney disease, bladder dysfunction, bladder calculi and previous or present cancer. Regarding study design, no study blinded participants to their allotted procedure except Pisco et al.,^17^ who compared PAE to sham. This lack of blinding is likely due to the surgical nature of the interventions since concealing may be impractical for interventions. The HoLEP group had a single trial which used double blinding.^19^ The largest sample size in the PAE study group was Gao et al., with 114 patients. In the HoLEP group, it was by Kuntz et al^21^ with 200 patients. The longest follow‐up time in the PAE group of studies was 24 months,^12^ representing a current limitation in evaluating the long‐term efficacy of PAE. In this study, the re‐treatment rate at 2 years for PAE was approximately 20%, a finding in line with a large cohort study and the UK‐ROPE study.^17^, ^31^ In comparison, the long‐term re‐treatment rates for HoLEP were reported to be about 5%.^32^

HoLEP was commonly performed using lasers in the 80‐ to 100‐W range, whereas most PAE studies used embolic agents in the 300‐ to 500‐μm size. PAE was performed via femoral access in all the included studies. Most RCTs on HoLEP were before 2016, and most PAE studies were published after 2015.

The baseline characteristics for most studies were similar (Tables 1A and 1B). The studies recorded various outcomes in addition to those selected for this review, such as IIEF‐5 score, PSA, and prostate volume. A variety of adverse effects (AE), such as strictures, contractures and mucosal damage, were seen in each study's TURP and HoLEP groups, whereas these AEs were absent in the PAE group (Tables 1C and 1D). This difference is not unexpected since PAE does not involve an invasive urethral component, unlike TURP and HoLEP.

## 4 DISCUSSION

The results of this meta‐analysis show that PAE and HoLEP are equivalent to TURP for improvement in IPSS, QoL and PVR at 3 and 12 months. At 3 months, TURP and HoLEP were unequivocally superior to PAE for improvement in Qmax. PAE is associated with lower SAE rates than surgical interventions like TURP and HoLEP. PAE and HoLEP have similar effects on the IPSS, QoL and PVR for 3 and 12 months.

The IPSS score improvement can be used to quantify the success or failure of BPH treatment.^40^ Prior literature has been variable regarding the efficacy of PAE, TURP and HoLEP on IPSS score outcomes, with some studies reporting non‐inferiority between the PAE‐TURP and HoLEP‐TURP.^1^, ^9^, ^33^, ^41^ A recent meta‐analysis reported that the effect size on IPSS score was MD 1.42 (95%CI: −0.92 to 3.75) when PAE and TURP were compared at 12 months [no statistically significant difference between treatments compared].^41^ Another meta‐analysis showed an IPSS effect size of MD 2.50 (95%CI: 0.78 to 4.21) in favour of TURP [inferiority of one of the treatments compared].^1^ When IPSS outcomes of TURP and HoLEP were compared, one meta‐analysis reported an effect size of MD −0.57 (95%CI: −1.80 to 0.67) in favour of HoLEP at 6 months [no statistically significant difference between treatments compared]. The same study reported an IPSS effect size of MD −0.78 (95%CI: −1.39 to −0.17) in favour of HoLEP (inferiority of one of the treatments compared) at 12 months.^34^ The present study reported that there was no statistically significant difference between PAE and HoLEP compared to TURP for improvement in IPSS. This study also reported that there is no statistically significant difference between PAE and HoLEP for improvement in IPSS. A notable trend here is that although PAE takes several months to achieve objective urodynamic results similar to surgical interventions, it does not lag in clinical symptom improvement. This observation is supported by the lack of significant difference between PAE in the subjective metrics at the 3‐month point (Figure 1A,B).

Regarding QoL score, one meta‐analysis comparing QoL outcomes between PAE and TURP reported an effect size of MD 0.21 (95%CI: −0.31 to 0.73) at 12 months [no statistically significant difference between treatments compared].^41^ Another meta‐analysis reported an effect size of MD 0.40 (95%CI: 0.09 to 0.71) at 12 months [inferiority of one of the treatments compared].^1^ The present study found no statistically significant difference between PAE and TURP in QoL outcomes at 3 and 12 months. There is sparse literature comparing pooled QoL outcomes between TURP and HoLEP. One meta‐analysis reported a WMD of −0.19 (95%CI: −0.68 to 0.30 at 3 months and WMD of −0.09 (95%CI: −0.65 to 0.47) at 12 months and showed no significant difference between HoLEP and TURP based on a random effects model. The present study reported no statistically significant difference between PAE compared to HoLEP for improvement at 3 and 12 months.

The PVR and uroflowmetry are non‐invasive urodynamic measures influenced by the mass of obstructing tissue removed in TURP or HoLEP and the amount of necrosis in PAE.^35^ One meta‐analysis comparing PVR outcomes between PAE and TURP reported an effect size of MD 21.16 (95%CI: −5.58 to 47.89) in favour of TURP at 12 months [no statistically significant difference between treatments compared].^41^ Another meta‐analysis investigating similar outcomes reported an effect size of MD 0.27 (95%CI: −2.08 to 3.00) [no statistically significant difference between treatments compared].^1^ A meta‐analysis investigated the effect of HoLEP vs. TURP on PVR in prostates < 100 g and reported an effect size of MD −9.93 (95%CI: −18.59 to −1.27) in favour of HoLEP [HoLEP was found to be superior to TURP]. The present study reported that there is no statistically significant difference between PAE and HoLEP TURP for improvement in PVR at 3 and 12 months. The present study also reported no statistically significant difference between PAE compared to HoLEP for improvement in PVR at 3 months and 12 months.

Prior meta‐analysis investigating the Qmax outcomes of PAE and TURP at 12 months reported an effect size of MD 3.78 (95%CI: 0.19 to 7.27), in favour of TURP [TURP was superior to PAE]. When HoLEP and TURP were compared for the same outcome, one study found an effect size of MD 1.26 (95%CI: −0.12 to 2.63) [no statistically significant difference between treatments compared] at 6 months and MD 1.46 (95%CI: 0.98 to 1.93) [HoLEP was superior to TURP] at 12 months, both in favour of HoLEP. The present study found that HoLEP and TURP were superior to PAE for Qmax improvement at 3 months. At 12 months, all three treatments had similar efficacy. Such a pattern is likely because HoLEP and TURP cause an immediate removal of the obstructing tissue (superiority at 3 months), whereas PAE requires several months to act and cause de‐obstruction (disappearance of superiority at 12 months).^14^ Abt et al. reported that PAE's effect on Qmax remains static from 12 to 24 months, whereas Qmax in the TURP cohort decreased.^12^

A correlation between post‐procedure prostate volume change and improvements in outcome measures has been established.^36^ The authors reported the prostate volume correlated with positive significant correlation between prostate volume and IPSS (*r* = 0.179, *p* = 0.002) and a negative significant correlation between prostate volume and Qmax (*r* = − 0.176, *p* = 0.003). These results are in line with this NMA, which showed more improvement in Qmax with HOLEP and TURP compared to PAE, whereas there were similar outcomes in other metrics as Qmax is directly influenced by the amount of prostate tissue resected.

Adverse events can be evaluated as aggregate (classified by severity) or risk ratios for individual adverse events. One meta‐analysis found no statistically significant difference in the adverse effects between PAE and TURP [OR 0.90; 95%CI: 0.20–4.05].^41^ The evidence for adverse events between HoLEP and TURP has been evaluated as risk ratios for individual adverse events.^9^, ^33^ The present study found that PAE and TURP were associated with lower adverse effect rates than HoLEP. The exclusion of Gao et al. from this analysis resulted in significant OR between PAE vs. TURP [OR:0.37 with 95%CI: between 0.15 and 0.91] and PAE vs. HoLEP. Based on this result, it is likely that PAE in the real world does have significantly lower SAE rates than TURP and HoLEP due to the minimally invasive nature of the procedure. Only Gao et al. and Radwan et al.^37^ have demonstrated a higher AE rate with PAE than with TURP, possibly due to underreporting of AE with TURP.^41^ The UK‐ROPE, a large observational study, found that 24% of PAE patients reported retrograde ejaculation compared to 47.5% of patients in the TURP arm. The same study reported similar incontinence rates between PAE and TURP (1% vs. 3%, respectively). The present study showed that TURP was associated with higher SAE rates than HoLEP, a finding which is in accordance with other quantitative syntheses comparing TURP and HoLEP.^9^, ^34^

PAE has also been associated with higher re‐treatment rates than TURP, which should be considered during patient selection.^10^ A recently published study showed a 10‐year retreatment rate of 23.8% after PAE.^5^ This increased re‐treatment rates might be attributable to new vascular channel formation, extensive collateral supply and regrowth of the prostatic stromal tissue. Retreatment rates are important to consider since they can significantly add to retreatment costs and patient morbidity.^38^ In view of the higher retreatment rate with PAE, it is prudent to counsel patients with life expectancies >5–10 years about the risk of LUTS recurrence and the risk associated with each procedure. It should be noted that the effects of multiple retreatment cycles have not been investigated.

PAE is associated with unique risks that are not associated with TURP and HoLEP. The need for fluoroscopic guidance and digital subtraction angiography (DSA), combined with the lengthy procedure time, especially during the learning curve, may cause significant radiation exposure with PAE. Zumstein et al.^39^ found minimal risk associated with PAE radiation exposure. Radiation exposure can be reduced by radial access and computed tomography (CT) angiography pre‐procedure with cone beam CT intra‐procedure to avoid multiple runs of DSA.^42^, ^43^

The evidence presented in this discussion from other studies is heterogeneous, with disagreement between results on various measures. This is partly due to the different inclusion criteria used for the quantitative analysis and a combination of different study designs in the same analysis. The present study includes only RCTs in the quantitative synthesis, possibly accounting for the divergence of results. This NMA can be helpful to policymakers as evidence for funding future research and clinicians for the appropriate choice of treatment for individual patients, especially for poor surgical candidates. This study suggests that PAE and HoLEP are effective alternatives to TURP at 3 and 12 months. PAE is likely associated with lower rates of serious adverse effects.

### 4.1 Limitations and future research

This NMA included only nine studies on PAE, with a pooled sample size of 536 patients. Since most studies comparing HoLEP and TURP were published before 2010, improvements in HoLEP technology/technique may not be represented. Using a random effects model for the evaluation may lead to more conservative estimates of the pooled effect size. There was a large amount of heterogeneity in most of the analyses conducted, which can be attributed to the combination of M‐TURP and B‐TURP into the same node, the different embolic agents used in PAE procedures, inconsistent inclusion/exclusion criteria for both PAE and HoLEP studies, different holmium laser devices, different power settings and energy sources used and differential operator preferences (Table S1A and Table S1B). Another factor to be considered during BPH treatment is the effect on sexual function. This NMA did not evaluate this outcome due to the lack of this outcome in the selected studies. Similarly, this NMA does not consider peri‐operative outcomes and their effect on patient selection.

Given that the majority of the studies included exclude prostate sizes >80 g, it is possible that the conclusions of this study may have limited generalizability to prostates >80 g. The majority of TURP procedures are done for prostates <80 g. The lack of procedures meant for larger prostates like open/robotic prostatectomy as comparators may also limit the conclusions drawn for prostates above 80 g.

Given the lack of long‐term data on PAE, the re‐treatment rates and long‐term comparative efficacy of PAE remain an open question. Future research may focus on more elaborate trials with longer follow‐up times to directly compare the efficacy and safety of PAE, HoLEP and TURP. At the time of writing, RCTs are comparing PAE and HoLEP head‐on.^44^ Standardized protocols implemented for multi‐centric trials for all three procedures may help reduce heterogeneity and reveal a more uniform effect size.

## 5 CONCLUSION

- There is no statistically significant difference between PAE and HoLEP for subjective measures of symptom improvement (IPSS and QoL) and PVR at 3 months and short‐term follow‐up of 1 year.
- Although PAE is associated with lesser improvement in Qmax than TURP and HoLEP at 3 months, it is also associated with lower rates of SAE.
- There is a lack of long‐term follow‐up data on PAE. The increased re‐treatment rates associated with PAE should also be considered when selecting patients for PAE. These results should be interpreted with caution, and each patient's case‐by‐case analysis should guide the choice of intervention.

## AUTHOR CONTRIBUTIONS

All authors contributed to the study's conception and design. All authors read and approved the final manuscript.

**Ansh Bhatia:** Methodology; data collection; formal analysis and investigation; writing—original draft preparation; review and editing. **Joao Gabriel Porto:** Writing—review and editing. **Aneesha Maini:** Data collection; methodology; formal analysis and investigation; writing—original draft preparation. **Deepak Langade:** Methodology; formal analysis and investigation. **Thomas R.W. Herrmann:** Writing—review and editing; supervision. **Hemendra Navinchandra Shah:** Writing—review and editing; supervision. **Shivank Bhatia:** Formal analysis and investigation; writing—review and editing; supervision.

## CONFLICT OF INTEREST STATEMENT

Shivank Bhatia reports consulting fees from Embolx Inc. Grant funding and Travel Support from Merit Medical. Stock ownership of East End LLC. The remaining authors report no competing financial interests.

## CONSENT FOR PUBLICATION

All authors agree with all the published material and consent for publication.

## Supporting information


**Figure S1:** PRISMA NMA Checklist of Items to Include When Reporting A Systematic Review Involving a Network Meta‐analysis.

**Figure S2:** PICOs representing the research question, participant population, intervention and outcome measure.

**Figure S3:** Study selection process.

**Figure S4:** Geometry of network. This visual represents the direct comparisons used to estimate the effect sizes of PAE vs HoLEP indirectly.

**Table S1A and S1B:** Inclusion, exclusion criteria and technical details of PAE and HoLEP studies.

## Data Availability

All data have been confirmed authentic by all the authors.
