# Supplementary material for: One‐year outcomes after prostate artery embolization versus laser enucleation: A network meta‐analysis
Source: BJUI Compass. 2023 Oct 27;5(2):189–206. doi: 10.1002/bco2.302 (PMC10869668; doi:10.1002/bco2.302)
Supplement: Supplementary file 1 — Figure S1: PRISMA NMA Checklist of Items to Include When Reporting A Systematic Review Involving a Network Meta‐analysis. [file BCO2-5-189-s002.docx]

**PRISMA NMA Checklist of Items to Include When Reporting A Systematic Review Involving a Network Meta-analysis**

| **Section/Topic** | **Item #** | **Checklist Item** | **Reported on Page #** |
| --- | --- | --- | --- |
| **TITLE** |  |  |  |
| Title | 1 | Identify the report as a systematic review *incorporating a network meta-analysis (or related form of meta-analysis).* | ***Title, page 1*** |
|  |  |  |  |
| **ABSTRACT** |  |  |  |
| Structured summary | 2 | Provide a structured summary including, as applicable:  **Background:** main objectives  **Methods:** data sources; study eligibility criteria, participants, and interventions; study appraisal; and *synthesis methods, such as network meta-analysis.*  **Results:** number of studies and participants identified; summary estimates with corresponding confidence/credible intervals; *treatment rankings may also be discussed. Authors may choose to summarize pairwise comparisons against a chosen treatment included in their analyses for brevity.*  **Discussion/Conclusions:** limitations; conclusions and implications of findings.  **Other:** primary source of funding; systematic review registration number with registry name. | Page 1-2 |
|  |  |  |  |
| **INTRODUCTION** |  |  |  |
| Rationale | 3 | Describe the rationale for the review in the context of what is already known*, including mention of why a network meta-analysis has been conducted.* | ***Page 3*** |
| Objectives | 4 | Provide an explicit statement of questions being addressed, with reference to participants, interventions, comparisons, outcomes, and study design (PICOS). | Page 4, supplemental figure |
|  |  |  |  |
| **METHODS** |  |  |  |
| Protocol and registration | 5 | Indicate whether a review protocol exists and if and where it can be accessed (e.g., Web address); and, if available, provide registration information, including registration number. | Page 4 |
| Eligibility criteria | 6 | Specify study characteristics (e.g., PICOS, length of follow-up) and report characteristics (e.g., years considered, language, publication status) used as criteria for eligibility, giving rationale. *Clearly describe eligible treatments included in the treatment network, and note whether any have been clustered or merged into the same node (with justification).* | ***Supplemental material, figure 2, table 2, page 4*** |
| Information sources | 7 | Describe all information sources (e.g., databases with dates of coverage, contact with study authors to identify additional studies) in the search and date last searched. | Page 4 |
| Search | 8 | Present full electronic search strategy for at least one database, including any limits used, such that it could be repeated. | Supplemental figure 1B |
| Study selection | 9 | State the process for selecting studies (i.e., screening, eligibility, included in systematic review, and, if applicable, included in the meta-analysis). | Supplemental figure 3 |
| Data collection process | 10 | Describe method of data extraction from reports (e.g., piloted forms, independently, in duplicate) and any processes for obtaining and confirming data from investigators. | Page 4 and 5 |
| Data items | 11 | List and define all variables for which data were sought (e.g., PICOS, funding sources) and any assumptions and simplifications made. | Page 4 |
| **Geometry of the network** | **S1** | Describe methods used to explore the geometry of the treatment network under study and potential biases related to it. This should include how the evidence base has been graphically summarized for presentation, and what characteristics were compiled and used to describe the evidence base to readers. | ***Supplemental figure 4*** |
| Risk of bias within individual studies | 12 | Describe methods used for assessing risk of bias of individual studies (including specification of whether this was done at the study or outcome level), and how this information is to be used in any data synthesis. | NIH quality assessment tool |
| Summary measures | 13 | State the principal summary measures (e.g., risk ratio, difference in means). *Also describe the use of additional summary measures assessed, such as treatment rankings and surface under the cumulative ranking curve (SUCRA) values, as well as modified approaches used to present summary findings from meta-analyses.* | Page 5 |
| Planned methods of analysis | 14 | Describe the methods of handling data and combining results of studies for each network meta-analysis. This should include, but not be limited to:   - *Handling of multi-arm trials;* - *Selection of variance structure;* - *Selection of prior distributions in Bayesian analyses; and* - *Assessment of model fit.* | Page 5 |
| **Assessment of Inconsistency** | **S2** | Describe the statistical methods used to evaluate the agreement of direct and indirect evidence in the treatment network(s) studied. Describe efforts taken to address its presence when found. | Page 5 |
| Risk of bias across studies | 15 | Specify any assessment of risk of bias that may affect the cumulative evidence (e.g., publication bias, selective reporting within studies). | Page 5 |
| Additional analyses | 16 | Describe methods of additional analyses if done, indicating which were pre-specified. This may include, but not be limited to, the following:   - Sensitivity or subgroup analyses; - Meta-regression analyses; - *Alternative formulations of the treatment network; and* - *Use of alternative prior distributions for Bayesian analyses (if applicable).* | ***NA*** |
|  |  |  |  |
| **RESULTS†** |  |  |  |
| Study selection | 17 | Give numbers of studies screened, assessed for eligibility, and included in the review, with reasons for exclusions at each stage, ideally with a flow diagram. | Supplementary figure 3 |
| **Presentation of network structure** | **S3** | Provide a network graph of the included studies to enable visualization of the geometry of the treatment network. | ***Supplemental figure 4*** |
| **Summary of network geometry** | **S4** | Provide a brief overview of characteristics of the treatment network. This may include commentary on the abundance of trials and randomized patients for the different interventions and pairwise comparisons in the network, gaps of evidence in the treatment network, and potential biases reflected by the network structure. | ***Pages 9, 10, 15*** |
| Study characteristics | 18 | For each study, present characteristics for which data were extracted (e.g., study size, PICOS, follow-up period) and provide the citations. | Supplemental table 1-3 |
| Risk of bias within studies | 19 | Present data on risk of bias of each study and, if available, any outcome level assessment. | 3 |
| Results of individual studies | 20 | For all outcomes considered (benefits or harms), present, for each study: 1) simple summary data for each intervention group, and 2) effect estimates and confidence intervals. *Modified approaches may be needed to deal with information from larger networks.* | ***Figures 1-5*** |
| Synthesis of results | 21 | Present results of each meta-analysis done, including confidence/credible intervals. *In larger networks, authors may focus on comparisons versus a particular comparator (e.g. placebo or standard care), with full findings presented in an appendix. League tables and forest plots may be considered to summarize pairwise comparisons.* If additional summary measures were explored (such as treatment rankings), these should also be presented. | ***Figures 1-5*** |
| **Exploration for inconsistency** | **S5** | Describe results from investigations of inconsistency. This may include such information as measures of model fit to compare consistency and inconsistency models, or summary of inconsistency estimates from different parts of the treatment network. | ***Figures 1-5*** |
| Risk of bias across studies | 22 | Present results of any assessment of risk of bias across studies for the evidence base being studied. | ***Figures 1-5*** |
| Results of additional analyses | 23 | Give results of additional analyses, if done (e.g., sensitivity or subgroup analyses, meta-regression analyses*, alternative network geometries studied, alternative choice of prior distributions for Bayesian analyses,* and so forth). |  |
|  |  |  |  |
| **DISCUSSION** |  |  |  |
| Summary of evidence | 24 | Summarize the main findings, including the strength of evidence for each main outcome; consider their relevance to key groups (e.g., healthcare providers, users, and policy-makers). | Page 11 |
| Limitations | 25 | Discuss limitations at study and outcome level (e.g., risk of bias), and at review level (e.g., incomplete retrieval of identified research, reporting bias). *Comment on the validity of the assumptions, such as transitivity and consistency. Comment on any concerns regarding network geometry (e.g., avoidance of certain comparisons).* | Page 15 |
| Conclusions | 26 | Provide a general interpretation of the results in the context of other evidence, and implications for future research. | Page 16 |
|  |  |  |  |
| **FUNDING** |  |  |  |
| Funding | 27 | Describe sources of funding for the systematic review and other support (e.g., supply of data); role of funders for the systematic review. This should also include information regarding whether funding has been received from manufacturers of treatments in the network and/or whether some of the authors are content experts with professional conflicts of interest that could affect use of treatments in the network. | ***Page 16*** |

PICOS = population, intervention, comparators, outcomes, study design.

* Text in italics indicateS wording specific to reporting of network meta-analyses that has been added to guidance from the PRISMA statement.

† Authors may wish to plan for use of appendices to present all relevant information in full detail for items in this section.

Supplementary item 1B: Search Strategy:

**Pubmed** - PAE search - 377 results.

(("prostatic hyperplasia"[MeSH Terms] OR benign prostatic hyperplasia[Text Word] OR "LUTS" OR "Lower urinary tract symptoms" OR "Urinary tract symptoms")) AND ("prostate artery embolization*"[All Fields] OR "prostat* arteries embolization*"[All Fields] OR "embolization of prostate arter*"[All Fields] OR "PAE"[All Fields] OR "embolization of the prostate arter*"[All Fields] OR "prostate artery embolization"[All Fields] OR "prostate arteries embolization"[All Fields] OR "embolization of prostate arter*"[All Fields] OR "embolization of the prostate arter*"[All Fields] OR "arterial embolization"[All Fields] OR "embolization of arter*"[All Fields] OR "prostat* artery embolisation*"[All Fields] OR "prostate arteries embolisation*"[All Fields] OR "embolisation of prostate arter*"[All Fields] OR "embolisation of the prostate arter*"[All Fields] OR "prostate artery embolisation"[All Fields] OR "prostate arteries embolisation"[All Fields] OR "embolisation of prostatic arter*"[All Fields] OR "embolisation of the prostate arter*"[All Fields])

**Pubmed** – HoLEP search - 912 results

(("Laser enucleation"[All fields] OR "HoLEP"[All fields] OR "enucleation of prostate"[All fields] OR "Prostate enucleation"[All fields] OR "Holmium Laser Enucleation"[All fields] OR "Prostate laser enucleation"[All fields] OR "Holmium Laser Enucleation of prostate")) AND ((("prostatic hyperplasia"[MeSH Terms] OR benign prostatic hyperplasia[Text Word] OR "LUTS" OR "Lower urinary tract symptoms" OR "Urinary tract symptoms")))

**Cochrane search** - 60 Trials matching – PAE

("prostatic hyperplasia" OR benign prostatic hyperplasia OR "LUTS" OR "Lower urinary tract symptoms" OR "Urinary tract symptoms") in Title Abstract Keyword AND ("prostate artery embolization*" OR "prostat* arteries embolization*" OR "embolization of prostate arter*" OR "PAE" OR "embolization of the prostate arter*" OR "prostate artery embolization" OR "prostate arteries embolization" OR "embolization of prostate arter*" OR "embolization of the prostate arter*" OR "arterial embolization" OR "embolization of arter*" OR "prostat* artery embolisation*" OR "prostate arteries embolisation*" OR "embolisation of prostate arter*" OR "embolisation of the prostate arter*" OR "prostate artery embolisation" OR "prostate arteries embolisation" OR "embolisation of prostatic arter*" OR "embolisation of the prostate arter*") in Title Abstract Keyword - (Word variations have been searched)

**Cochrane search** – 248 trials matching – HoLEP - 248 Trials**:** matching ("Laser enucleation" OR "HoLEP" OR "enucleation of prostate" OR "Prostate enucleation" OR "Holium Laser Enucleation" OR "Prostate laser enucleation" OR "Holium Laser Enucleation of prostate") in Title Abstract Keyword AND ("prostatic hyperplasia" OR benign prostatic hyperplasia OR "LUTS" OR "Lower urinary tract symptoms" OR "Urinary tract symptoms") in Title Abstract Keyword - (Word variations have been searched),.

**EMBASE Search** – 292 Results - PAE

[[Prostate [or] prostate artery] [and] embolization] [and] [urination [or] benign prostatic hyperplasia]

**EMBASE Search** – 53 results – HoLEP search

[[Prostate [or] prostate artery] [and] [urination [or] benign prostatic hyperplasia]] [and] [laser [and] enucleation]

Total Search results – 1942.

(Supplementary item ends)

Title of Manuscript: 1 year Outcomes after Prostate Artery Embolization versus Laser enucleation: A Network Meta-Analysis

Journal Name: British Journal of Urology International
