# Supplementary material for: One‐year outcomes after prostate artery embolization versus laser enucleation: A network meta‐analysis
Source: BJUI Compass. 2023 Oct 27;5(2):189–206. doi: 10.1002/bco2.302 (PMC10869668; doi:10.1002/bco2.302)
Supplement: Supplementary file 2 — Figure S2: PICOs representing the research question, participant population, intervention and outcome measure. [file BCO2-5-189-s003.docx]

**Eligibility Criteria**:

PICOs

| **Participants** | **Intervention** | **Comparator** | **Outcomes** | **Description of Outcome** |
| --- | --- | --- | --- | --- |
| Adult males with moderate to severe LUTS due to BPH | PAE  HoLEP | TURP* | International Prostate Symptom Score (IPSS) | Assesses the quality of life or bother score based on the patient’s perception of the problem. Scored from 0-35 |
|  |  |  | IPSS-Quality of Life Score (QoL) | A comprehensive score determining the impact of BPH symptoms on the patient’s quality of life. Scored from 0-6. Part of the IPSS standard questionnaire but used as an independent criterion for LUTS evaluation. |
|  |  |  | Post-void Residual volume (PVR) | Urodynamic metric which measures urine (ml) left in the bladder after an average micturition. |
|  |  |  | Maximum urinary flow velocity (Q max) | Purely urodynamic metric which determines maximum urinary flow velocity achieved in micturition. |
|  |  |  | Serious Adverse effects (Dindo-Clavien Classification grade >3) | Dindo-Clavien classification > 3 are Adverse effects which require surgical, radiological or endoscopic interventions for correction (13). |

*Bipolar TURP and Monopolar TURP were combined in the same node due to similar efficacies and indirect comparison (PAE vs HoLEP) nature of this study.

Title of Manuscript: 1 year Outcomes after Prostate Artery Embolization versus Laser enucleation: A Network Meta-Analysis

Journal Name: British Journal of Urology International
