# Supplementary material for: One‐year outcomes after prostate artery embolization versus laser enucleation: A network meta‐analysis
Source: BJUI Compass. 2023 Oct 27;5(2):189–206. doi: 10.1002/bco2.302 (PMC10869668; doi:10.1002/bco2.302)
Supplement: Supplementary file 3 — Figure S3: Study selection process. [file BCO2-5-189-s005.docx]

**Identification of studies via databases and registers**

Records removed *before screening*:

Duplicate records removed (n = 282) via Excel

Records identified from EMBASE, PUBMED and Cochrane: 1942

**Identification**

Records screened

(n = 1660)

Records excluded**

(n = 1552)

Reports sought for retrieval

(n = 108)

Reports not retrieved

(n =0)

**Screening**

Reports excluded:

Different indication/outcome (n = 26)

Ongoing trial/ not published (n = 3)

Irrelevant

(n = 59)

Reports assessed for eligibility

(n = 108)

Studies included in systematic review

(n = 20)

Studies included in meta-analysis

(n = 18)

**Included**

** Studies which were obviously irrelevant, non-randomized trials, case series, cost analysis, studies without even one of the outcome variables under investigation, case reports, observational studies, retrospective studies.

Title of Manuscript: 1 year Outcomes after Prostate Artery Embolization versus Laser enucleation: A Network Meta-Analysis

Journal Name: British Journal of Urology International
