# Supplementary material for: One‐year outcomes after prostate artery embolization versus laser enucleation: A network meta‐analysis
Source: BJUI Compass. 2023 Oct 27;5(2):189–206. doi: 10.1002/bco2.302 (PMC10869668; doi:10.1002/bco2.302)
Supplement: Supplementary file 4 — Figure S4: Geometry of network. This visual represents the direct comparisons used to estimate the effect sizes of PAE vs HoLEP indirectly. [file BCO2-5-189-s004.docx]

Title of Manuscript: 1 year Outcomes after Prostate Artery Embolization versus Laser enucleation: A Network Meta-Analysis

Journal Name: British Journal of Urology International.
