# Supplementary material for: One‐year outcomes after prostate artery embolization versus laser enucleation: A network meta‐analysis
Source: BJUI Compass. 2023 Oct 27;5(2):189–206. doi: 10.1002/bco2.302 (PMC10869668; doi:10.1002/bco2.302)
Supplement: Supplementary file 5 — Table S1A and S1B: Inclusion, exclusion criteria and technical details of PAE and HoLEP studies. [file BCO2-5-189-s001.docx]

| **Study name (Country)** | **Inclusion criteria** | **Exclusion criteria** | **Blinding** | **Embolic particles used (PAE)** | **Vascular access site** | **Type of TURP used** | **Longest Follow-up** |
| --- | --- | --- | --- | --- | --- | --- | --- |
| Abt, 2021 (Switzerland) | Refractory BPH-LUTS; Age &gt;40 year; IPSS PAE:48 &gt; 8; QOL 3; prostate size 25-80 ml; TURP:51 candidate for TURP; refractory to medical therapy or refuse to consider (further) medical treatment; Qmax &lt; 12 ml/s and/or urinary retention; written informed consent | Severe atherosclerosis, aneurysmatic changes or severe tortuosity in the aortic bifurcation or internal iliac arteries, a-contractile detrusor, neurogenic lower urinary tract dysfunction, urethral stenosis, bladder diverticulum, bladder stone, allergy to intravenous contrast media, contraindication for magnetic resonance imaging, pre-interventionally proven carcinoma of the prostate, and glomerular filtration rate <60 ml/min. | None | Embozene microspheres (250–400mm; Boston Scientific, Natick, MA, USA) | Femoral | M-TURP | **24 months** |
| Abt, 2018 (Switzerland) | Age ≥ 40 years, IPSS ≥ 8, Qol ≥ 3, Qmax < 12 ml/s or urinary retention, PV 25–80 ml, ineffective or refuse medical treatment | Severe artery-related disease, a-contractile detrusor, neurogenic lower urinary tract dysfunction, urethral stenosis, bladder diverticulum, bladder stone, renal failure, prostate cancer | None | 250–400-μm microspheres | Femoral | M-TURP | 3 months |
| Gao, 2014 (China) | Failed medical treatment with 2 weeks washout, IPSS > 7, Qmax < 15 ml/s, PV 20–100 ml. | Detrusor hyperactivity or hypo-contractility, urethral stricture, prostate cancer, diabetes mellitus, and previous prostate, bladder neck, or urethral surgery. | None | Polyvinyl alcohol microspheres (355–500 mm in diameter, Avalon; Cook) | Femoral | B-TURP | 24 months |
| Zhu, 2018 (China) | BPH-LUTS patients without a history of previous surgery, without taking alpha-blockers for 4 weeks. |  | None | 100-300um and 300-500um microspheres | Femoral | TURP |  |
| Insausti, 2020 (Spain) | Age > 60 years, IPSS ≥ 8, Qol ≥ 3, Qmax ≤ 10 ml/s or urinary retention, symptoms refractory to medical therapy for > 6 months. | Severe artery-related disease, non-visualization of the prostatic artery, urethral stenosis, detrusor failure or neurogenic bladder, renal failure, prostate cancer. | None | 300-500 um microspheres | Femoral | B-TURP | 12 months |
| Carnavale 2016 (Brazil) | Age ≥ 45 years, Refractory to medical therapy for > 6 months, IPSS > 19, Qmax ≤ 12 ml/s, PV 30–90 ml. | Renal failure, bladder calculi or diverticula, suspected prostate cancer, urethral stenosis, or neurogenic bladder disorders | None | 300-500 um microspheres | Femoral | M-TURP | 12 months |
| Radwan 2020 (Egypt) | IPSS > 8, average flow < 10 mL/s, PV <100 ml | Elevated kidney functions, prostate cancer, history of prostatic or urethral operations, bladder diverticulum, urinary tract infection | None | Microspheres | Femoral | M-TUPR and B-TURP | 6 months |
| Yoshinaga et al. 2014 (China) | men >50 years old; (IPSS) >19 refractory to medical treatment; negative screening for prostate cancer; prostate volume between 30-90g on magnetic resonance image (MRI); and bladder outlet obstruction confirmed by urodynamic examination | Renal failure, bladder calculi or None diverticulum, or urethral stenosis | None | NR | Femoral | NR | 18 months |
| Pisco 2020 (Portugal) | Males over 45 years old; diagnosis of LUTS/BPH based on clinical history, digital rectal examination, urinalysis, trans-rectal ultrasound (TRUS), and prostate-specific antigen (PSA); severe LUTS defined in screening and a baseline visit 2 weeks apart, by an IPSS of 20 and a QoL score of 3 after a minimum of 6- month treatment with alpha-blockers for LUTS/BPH; Qmax <12 ml/s; PV 40 cm3 | Computed Tomography (CT) angiography showing that prostatic arteries were not feasible for PAE; previous surgical or invasive prostate treatments such as TURP, transurethral microwave therapy, transurethral needle ablation, laser, or any other minimally invasive treatment; acute, chronic prostatitis or suspected prostatitis including chronic pain, intermittent pain, or abnormal sensation in the penis, testis, anal, or pelvic area in the previous 12 months; history of prostate or bladder cancer or pelvic irradiation; or recurrent urinary tract infections (more than one episode in the previous 12 months); history of neurogenic bladder LUTS secondary to neurologic disease; advanced atherosclerosis and tortuosity of iliac and prostatic arteries; secondary renal insufficiency (due to prostatic obstruction); large bladder diverticula or stones; detrusor failure; bleeding disorders, previous medication use. | Single blind | Bead Block (BTG plc, London, UK) 300-500 mm | Femoral | NA | 12 months |
| Ray 2018 (United Kingdom) | Men with LUTS who had consented to undergo PAE, TURP, open prostatectomy, or holmium enucleation of the prostate at 1 of the United Kingdom Register of Prostate Embolization collaborating centres | Not set* | None | NR | Femoral | NR | 12 months |
| NR = Not Reported | *Since this was an observational study, the authors deliberately did not set stringent exclusion criteria to mimic real-world conditions. | | | | | |  |

| Study name (Country) | Inclusion criteria | Exclusion criteria | Blinding | Laser details (HoLEP) (Power and model) |
| --- | --- | --- | --- | --- |
| \| Sun et al., 2014  (China) \| \| --- \| | Age <90 years old with no contraindication to surgery; Qmax less than or equal to 10 ml/s; PVR greater than or equal to 50 ml; IPSS greater than or equal to 8; prostate weight less than 100 grams as determined by transrectal ultrasonography | Treatment with transurethral prostate surgery previously; neurogenic bladder; suspected prostate cancer | Double-blinded. | F26 OLYMPUS holmium laser prostate rectoscope with The 550-lm holmium laser fibre was connected, and the output power was 98 W |
| Fayad et al., 2015  (Egypt) | All male patients with bothersome lower urinary tract symptoms due to BPH with indications for surgical intervention regardless of the patient’s age, IPSS, and prostatic size | Patients with mild symptoms (IPSS<8 and maximum urinary flow rate greater than or equal to 15 mL/s and minimal postvoiding residual urine), small adenomas less than 20g measured by transrectal ultrasound, urethral stricture, neurogenic bladder, vesicoureteric reflux, huge retentive bladder diverticulum, previous prostatic surgeries, prostatic adenocarcinoma, patients receiving anticoagulant drugs | None | 100 W holmium yttrium aluminum garnet laser (Device Lumenis, Medical Systems, Santa Clara, CA). |
| Kuntz et al., 2004  (Germany) | AUA symptom score 12 or greater, peak urinary flow rate 12 ml per second or less (voided volume above 50 ml, urodynamic obstruction in pressure flow studies, and prostate volume less than 100 ccs, as estimated by transrectal ultrasound. | Cases of carcinoma of the prostate (where patients had an abnormal digital rectal examination, elevated serum PSA, and suspicious lesions on TRUS). Patients who had undergone previous urethral or prostatic surgery were also excluded. | None | A maximum average power of 80 W (2.0 J at 40 Hz) or 100 W (2.0 J at 50 Hz). |
| Gupta et al., 2005  (India) | Patients who were candidates for TURP and with glands of greater than 40g | Any patient with a previous history of prostatic and urethral surgery, neurovesical dysfunction, and carcinoma of the prostate was excluded | None | 550-µm end- firing laser fibre and a 100 W holmium-YAG laser source (Coherent Inc., Santa Clara, CA, USA). Power settings were 80–100 W at 2–1.5 J/ s and 50–40 Hz. |
| Montorsi/Rigatti et al. 2004 (Italy) | Patients with BPH and documented obstructed voiding symptoms in whom previous pharmacological therapy had failed; younger than age 75 years, peak urinary flow rate less than 15 ml per second, post-void residual urine less than 100 ccs, medical therapy failure, transrectal ultrasound adenoma volume less than 100 grams and urodynamic obstruction | Neurogenic bladder, a diagnosis of prostate cancer and any previous prostatic, bladder neck, or urethral surgery | None | The holmium laser energy was delivered by a 360 um fibre placed in a 24F resectoscope. Enucleation was performed at 2.0 J and 35 Hz |
| Tan et al., 2003 (New Zealand)/  Liam C et al., 2006 (NZ) | Prostate volume, as calculated by a TRUS volume of 40 to 200 ml, Qmax 15 ml/second or less, AUA symptom score 8 or greater, post-void residual less than 400 ml and Schafer grade 2 or greater. | TRUS-guided needles were performed to exclude prostate carcinoma cases from the study. Catheterized patients and those with a history of urethral or prostatic surgery were also excluded | None | (60 to 100 W) Ho laser (Lumenis, Tel Aviv, Israel) with a maximum average power of 100 W (2.0 J at 50 Hz).  Wavelength: 2,140 nm with maximum power was set at 100 W for each case |
| Basić et al.,  (Serbia), 2013 | postvoid residue=50ml, prostate volume up to 50g, repeated episodes of acute urinary retention, indwelling urinary catheter, recurrent urinary tract infection, recurrent hematuria due to BPH and IPSS score >19. | Voiding disorders out of BPH origin, previous urethral, bladder, neck or prostatic surgery, and history of prostate cancer | None | 2J/50Hz, 100-watt holmium laser device (VersaPulse® PowerSuite, Lumenis, Yokneam, Israel), using a 550-m end  Firing fiber (SlimLineTM 550, Lumenis Inc.) |
| Fayad et al., 2011  (Egypt) | Presented with LUTS due to BPH and in whom surgery was indicated | Patients with mild symptomatology: IPSS <8, Qmax >15ml/sec, and minimal postvoid residual urine. ; patients with small prostates (<20g), patients with urethral stricture, neurogenic bladder, vesicoureteral reflux, huge retentive bladder diverticulum in whom open surgery to remove the diverticulum was preferred, history of urethral or prostatic surgeries, prostate cancer, receiving anticoagulant therapy | None | 100W holmium: yttrium-aluminium-garnet laser device (Lumenis Medical Systems, Santa Clara, CA). A 550 l |
| Gilling et al., (2012) | TRUS volume of 40-200mL, Qmax less than or equal to 15 mL/s or less, AUA symptoms score greater than or equal to 8, PVR less than 400 mL, and Schafer grader greater than or equal to 2. | Patients who had undergone previous prostatic or urethral surgery, who had had carcinoma of the prostate or who were in urinary retention were excluded | None | (60 to 100 W) Ho laser (Lumenis, Tel Aviv, Israel) with a maximum average power of 100 W (2.0 J at 50 Hz) |
| Jhanwar et al. (2006) (India) | Age younger than 75 years after failed or poor response to medical therapy, Qmax <15 ml/s, prostatic size of more than 60 g, gross hematuria secondary to BPH, recurrent urinary tract infection (UTI), acute urinary retention, postvoid residual (PVR) more than 150 ml, and Schafer Grade II or more in pressure flow study | Associated neurogenic bladder, stricture urethra, carcinoma prostate, or previous history of intervention. | None | End firing holmium laser fibre (550 µm, versa power suit) with power setting (2 J at 40–50 Hz, 80–100 W). |

Title of Manuscript: s year Outcomes after Prostate Artery Embolization versus Laser enucleation: A Network Meta-Analysis

Journal Name: British Journal of Urology International
